# Retrograde Inferior Vena caval Perfusion for Total Aortic arch Replacement Surgery (RIVP-TARS): study protocol for a multicenter, randomized controlled trial

**DOI:** 10.1186/s13063-019-3319-2

**Published:** 2019-04-24

**Authors:** Jing Lin, Zhaoxia Tan, Hao Yao, Xiaolin Hu, Dafa Zhang, Yuan Zhao, Jiyue Xiong, Bo Dou, Xueshuang Zhu, Zhong Wu, Yingqiang Guo, Deying Kang, Lei Du

**Affiliations:** 1 Department of Anesthesiology, West China Hospital, Sichuan University, No. 37 Guo Xue Alley, Chengdu, 610041 Sichuan Province China; 2 Cardiovascular Center of the Second Affiliated Hospital, Nanjing Medical University, No. 121, Jiangjiaruan Road, Gulou District, Nanjing, 210000 Jiangsu Province China; 3 Department of Anesthesiology, First Affiliated Hospital of University of South China, No. 151, Yanjiang West Road, Yuexiu District, Guangzhou, 510000 Guangdong Province China; 4 Department of Thoracic Cardiovascular Surgery, First Affiliated Hospital, Wannan Medical University, No. 2, Chushan West Road, Jinghu District, Wuhu, 230000 Anhui Province China; 5 Department of Cardiovascular Surgery, The Second Xiangya Hospital, Central South University, No. 139, People’s Road, Furong District, Changsha, 410000 Hunan Province China; 6 Department of Cardiovascular Surgery, West China Hospital, Sichuan University, No. 37, Guo Xue Alley, Chengdu, 610041 Sichuan Province China; 7 Department of Evidence-based Medicine and Clinical Epidemiology, West China Hospital, Sichuan University, No. 37, Guo Xue Alley, Chengdu, 610041 Sichuan Province China

**Keywords:** Type A aortic dissection, Hypothermia, Cardiac arrest, Antegrade cerebral perfusion, Retrograde inferior vena caval perfusion

## Abstract

**Background:**

During total aortic arch replacement surgery (TARS) for patients with acute type A aortic dissection, the organs in the lower body, such as the viscera and spinal cord, are at risk of ischemia even when antegrade cerebral perfusion (ACP) is performed. Combining ACP with retrograde inferior vena caval perfusion (RIVP) during TARS may improve outcomes by providing the lower body with oxygenated blood.

**Methods:**

This study is designed as a multicenter, computer-generated, randomized controlled, assessor-blind, parallel-group study with a superiority framework in patients scheduled for TARS. A total of 636 patients will be randomized on a 1:1 basis to a moderate hypothermia circulatory arrest (MHCA) group, which will receive selective ACP with moderate hypothermia during TARS; or to an RIVP group, which will receive the combination of RIVP and selective ACP under moderate hypothermia during TARS. The primary outcome will be a composite of early mortality and major complications, including paraplegia, postoperative renal failure, severe liver dysfunction, and gastrointestinal complications. All patients will be analyzed according to the intention-to-treat protocol.

**Discussion:**

This study aims to assess whether RIVP combined with ACP leads to superior outcomes than ACP alone for patients undergoing TARS under moderate hypothermia. This study seeks to provide high-quality evidence for RIVP to be used in patients with acute type A aortic dissection undergoing TARS.

**Trial registration:**

Clinicaltrials.gov, ID: NCT03607786. Registered on 30 July 2018.

**Electronic supplementary material:**

The online version of this article (10.1186/s13063-019-3319-2) contains supplementary material, which is available to authorized users.

## Background

Acute type A aortic dissection (AAAD) is a life-threatening condition involving tearing of the intimal layer in the ascending aorta, greater curve of the aortic arch, and proximal descending thoracic aorta. AAAD is one of the most serious cardiovascular events, with mortality rates of 50% within the first 48 h and 75% within 2 weeks of the onset of symptoms [1–3]. It requires immediate total aortic arch replacement surgery (TARS), in which both the ascending aorta and aortic arch are replaced by artificial vascular grafts. If necessary, a transaortic stent elephant trunk may also be implanted [4].

Because opening the distal anastomosis is required during reconstruction of the aortic arch, patients may undergo a prolonged period of circulatory arrest in order to provide clinicians with a clear operating field. To minimize potential ischemic injury to organs, patients are placed under deep hypothermic circulatory arrest (DHCA), first reported by Griepp and colleagues in 1975 [5]. DHCA is considered safe for periods of only 20–30 min at 14–20 °C [6], but it is associated with high rates of mortality (13–40%) [7, 8], stroke (7–30%) [8, 9], and postoperative acute kidney injury (40–50%) [10, 11]. The risk of such events increases with DHCA duration. Furthermore, the low body temperature during DHCA can activate platelets, and the extended cardiopulmonary bypass (CPB) time for cooling and rewarming necessitates greater use of blood products than other cardiac surgeries [12, 13]. This has led clinicians to develop adjunct techniques that, when combined with DHCA, can improve patient outcomes; these techniques include retrograde cerebral perfusion (RCP) and selective antegrade cerebral perfusion (ACP) under mild-to-moderate hypothermic circulatory arrest (MHCA).

RCP involves retrograde perfusion of cold, oxygenated blood into the superior vena cava, which maintains the cerebral oxygen supply and is thought to flush out the atherosclerotic debris and air generated during the repair [14, 15]. However, studies have suggested that combining RCP and DHCA does not reduce risk of stroke and may actually increase the risk of temporary neurological dysfunction [16]. Furthermore, RCP demands a high volume of blood products [17].

The current preferred strategy for AAAD surgery is ACP + MHCA at 25–30 ^°^C [18, 19]. ACP is performed by the perfusion of oxygenated blood via the subclavian artery, innominate artery, or the right axillary artery during hypothermic circulatory arrest (HCA). Studies show that ACP + MHCA can significantly improve clinical outcomes, leading to lower rates of mortality and neurological deficit than RCP + DHCA [20, 21]. Nevertheless, ACP + MHCA is associated with overall 30-day mortality rates of 5.3–19% [22, 23] and stroke rates of 6.7–10% [24, 25]. The incidence of acute kidney injury in ACP + MHCA ranges from 19 to 54%, [26, 27] with 5–9% of these patients requiring renal replacement therapy, which is itself associated with an elevated short-term mortality rate of 30–75% [26, 28].

Since lower-body circulatory arrest is needed during ACP, the organs in the lower body, such as the viscera and spinal cord, are still at risk of ischemia. Moderate hypothermia is still required to extend the tolerance of organs to anoxia, which may prolong CPB duration for cooling and rewarming. Lower-body circulatory arrest may, therefore, be a direct factor contributing to postoperative adverse events. To reduce the risk of ischemic injury to these organs, the viscera and spinal cord should be well perfused during opening distal anastomosis.

Retrograde inferior vena caval perfusion (RIVP) is a strategy used to provide the lower body with oxygenated blood. No venous valves are present in the visceral vein [29], which makes RIVP possible. Previous animal studies have revealed that RIVP may benefit the abdominal organs by maintaining continuity in circulation and providing adequate blood flow for oxygen delivery [30]. However, the debate remains unsettled about the optimal RIVP pressure and the feasibility of simultaneous retrograde bi-caval perfusion at a nasopharyngeal temperature of 18 °C [31]. It has been reported that simultaneous continuous retrograde perfusion through both the inferior and superior venae cavae can protect organs at a rectal temperature of 20 ^°^C and perfusion flow rate of 300–600 mL/min [32]. We are unaware of subsequent studies focusing on RIVP.

The available evidence suggests that ACP provides more physiological blood flow to the brain than RCP, while RIVP reduces the risk of lower body ischemia by providing venous-to-arterial blood flow. Therefore, we have designed a trial to test whether the combination of ACP and RIVP is feasible under moderate hypothermia during reconstruction of the aortic arch. Furthermore, we will investigate the hypothesis that combining ACP and RIVP is superior to ACP alone for TARS under moderate hypothermia.

### Objective and hypothesis

The primary aim of this multicenter trial is to assess whether RIVP combined with ACP leads to better outcomes than ACP alone for patients undergoing TARS under moderate hypothermia (Protocol no. 1.0, dated 10 January 2018). We hypothesize that combining RIVP with ACP will lead to lower incidences of mortality, major complications and postoperative temporary neurological defects than ACP alone, as well as shorter duration of ventilation and stay in the intensive care unit (ICU). The results of this trial will serve as a foundation for future clinical recommendations regarding the application of RIVP in TARS and should potentially improve treatment.

## Methods

### Study design

This study is designed as a multicenter, randomized controlled, assessor-blind, parallel-group study with a superiority framework. Five clinical research centers in China will participate: West China Hospital of Sichuan University, Second Affiliated Hospital of Nanjing Medical University, First Affiliated Hospital of Wannan Medical University, The Second Xiangya Hospital of Central South University, and First Affiliated Hospital of the University of South China.

A total of 636 participants with type A aortic dissection will be randomly assigned by computer to either the MHCA (control) group or the RIVP group at a ratio of 1:1. Patients assigned to the control group will receive selective ACP alone under moderate hypothermia, while patients in the RIVP group will receive selective ACP combined with RIVP under moderate hypothermia. All patients will be admitted to a cardiovascular ICU after surgery, where they will remain until they are considered stable enough to transfer back to a general unit. Postoperative follow-up will be conducted throughout hospitalization (regardless of length of stay) and for up to 30 days after surgery if the patient is discharged, which will be sufficient to show the prognosis of surgical treatment. Patient enrollment began in January 2019 and is expected to be complete within 3 years thereafter. The following members of the study will be blinded to the patients’ group allocation: patients themselves, outcome assessors, and the statistician.

Perfusionists and surgeons in the trial will be experienced in performing the necessary procedures. Interventions will be carried out intraoperatively. Patients’ medical records will be reviewed for in-hospital complications and medication usage.

**Fig. 1:**
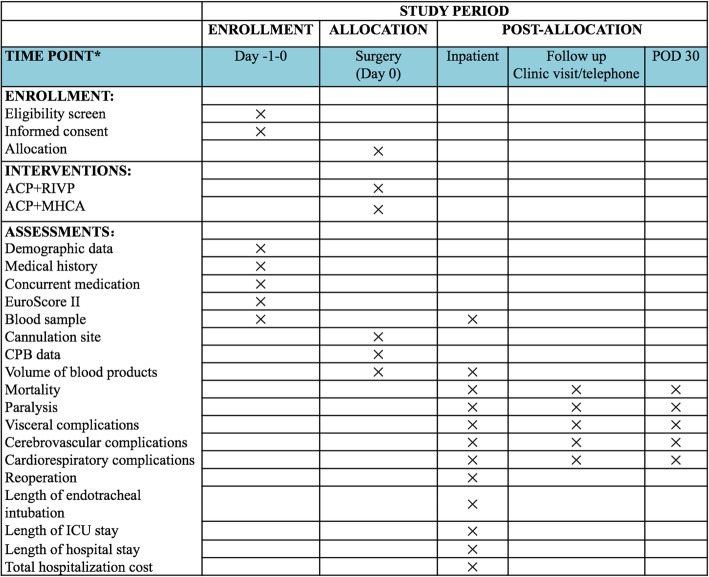
Schedule of enrollment, intervention, and assessment according to the Standard Protocol Items: Recommendations for Interventional Trials (SPIRIT) Statement. *ACP* selective antegrade cerebral perfusion, *MHCA* moderate hypothermic circulatory arrest, *RIVP* retrograde inferior vena caval perfusion, *POD* postoperative day, *EUROSCOREII* European System for Cardiac Operative Risk Evaluation II, *CPB* cardiopulmonary bypass, *ICU* intensive care unit

This protocol is designed in accordance with the Standard Protocol Items: Recommendations for Interventional Trials (SPIRIT) guidelines for interventional trials [33]. The SPIRIT Checklist is shown in Additional file 1, and the SPIRIT Figure is shown in Fig. 1. Participating centers will be required to sign a collaboration contract that lays out the responsibilities, intellectual property ownership, and publication processes. The funding for this trial covers only organizational costs and meetings; there is no third-party funding support for this trial. Changes to the protocol will be submitted to our Ethics Committee with a detailed description of the changes before going forward. All on-going severe adverse events (SAEs) will be followed up and documented until final outcomes are determined.

### Ethics and registration

**Fig. 2:**
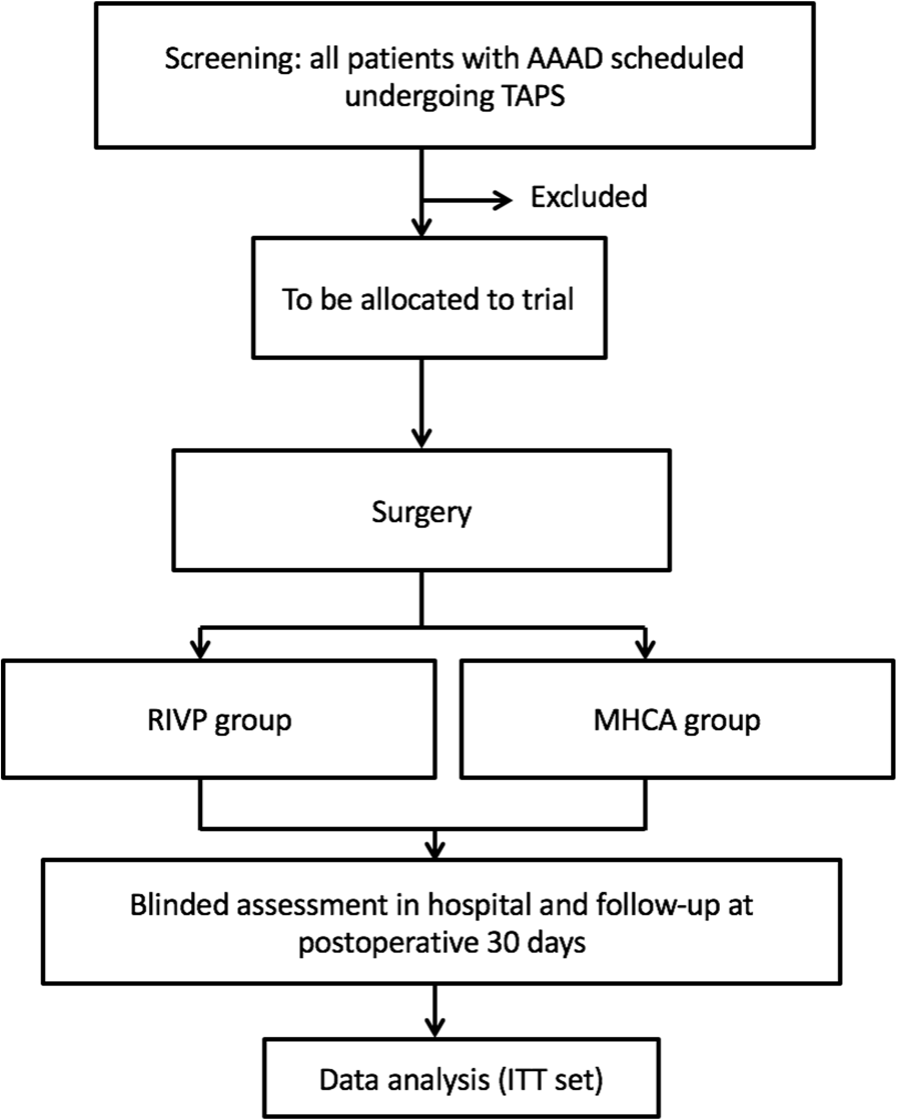
Flow chart of the clinical trial

The protocol of this study follows guidelines set by the Declaration of Helsinki and is in accordance with the Medical Research Involving Human Subjects Act (WMO) as well as Good Clinical Practice guidelines [34]. Central ethical approval has been confirmed from the Biomedical Ethics Committee of West China Hospital (ref approval no. 201824) and we will not begin recruiting at other centers in the trial until local ethical approval has been obtained. This trial has been registered at the Clinical Trial Registry (NCT03607786). All results will be presented in accordance with the Consolidated Standards of Reporting Trials (CONSORT) Statement (Fig. 2).

### Recruitment of study population

Recruitment began on 1 January 2019 and is expected to be complete within 36 months (31 December 2021). Participants present on surgical lists will be recruited from the emergency department (ED), the cardiovascular wards or cardiac ICU depending on the coordinating center. Patients transferred directly to the OR will be recruited in the ED. Patients transferred to the cardiac ICU or ward under sedation and blood pressure control will be recruited in the ICU. The surgery need not be postponed for obtaining consent, even if the patient is transferred directly to the OR. If a patient with AAAD in the ED requires emergency surgery, the surgical fellow will be informed and will inform the anesthesiologist, research assessors and perfusionist to obtain the relevant consents in the ED.

After surgical consent is obtained by surgical fellows, a researcher will clearly explain to patients or their relatives sufficient information about this trial, including the benefits and drawbacks associated with participation. Simple language will be used to facilitate the understanding of medical terms. Participants who give consent will provide demographic and general medical data as described below in the section “Baseline study visit.”

The target study population is all patients aged 18 years or older diagnosed with AAAD and scheduled for elective or emergency TARS under CPB. Patients are eligible for the study if they are older than 18 years old and show AAAD on contrast computed tomography. Patients will be excluded from the study if they are unable to understand or give informed consent, if they are pregnant or if they are already participating in another clinical trial that might interfere with the primary or secondary outcomes of the present trial. Patients will also be excluded if they have had a hemi-arch replacement or if they or their relatives revoke consent. Patients can leave the study at any time for any reason if they wish to do so without any consequences. Patients who withdraw from the study will not be replaced. Withdrawals will be documented in the electronic case report form (eCRF). The investigator can decide to withdraw a participant from the study for urgent medical reasons.

### Data collection and management

All data will be recorded on a paper-based case report form (CRF). After in-hospital data are recorded, a trained assessor will enter the clinical data from the paper CRF into a web-based database (http://edc.cd120.info:8081/Edc). All participating centers, as well as the principal investigator, will have 24-h access to the eCRFs. If data are entered incompletely or incorrectly, the principal investigator can contact the participating centers for further clarification. All outcome parameters will be recorded by a member of the trial team at each center at the time of enrollment and throughout the follow-up period. All trial data will be stored on a secure server at the Data Coordinating Center, which will remain confidential, and will be retained for 15 years after completion of the study (defined as 365 days after follow-up of the last patient) and will be anonymized if requested by the authorities. Parameters critical to the primary aim of this trial will be monitored remotely.

### Baseline study visit

As part of the baseline visit, we will collect patient information including age, height, weight, European System for Cardiac Operative Risk Evaluation (EuroSCORE II) [35], smoking and drinking status, diabetes mellitus, peripheral arterial disease, lipid profile, pulmonary and cardio-cerebral co-morbidity, and pulmonary infection during the preceding 30 days. Preoperative hematology and biochemistry assessments will also be performed including full blood count, electrolytes, liver and renal function tests, coagulation profile, thyroid function tests, and C-reactive protein. Enhanced thoracic and abdominal computed tomography will be performed to confirm the preoperative diagnosis, and preoperative transthoracic echocardiography will be used to measure left ventricular function as well as to detect valvular disease or other pathologies that meet the exclusion criteria. Additional clinical and study data will also be collected at baseline, as well as at other study time points (Fig. 1).

### Randomization and blinding

Patients will be randomly assigned to either the RIVP group or the control group by an independent statistician uninvolved in the trial using a computer-generated randomization list. The randomization will take place upon entry into the operating room (OR). The study is designed such that the outcome assessor and patient will be blinded. The principal investigator at each center will reveal the randomization to the perfusionist but will not perform the perfusion or collect or analyze the data. The perfusionist will perform RIVP, record RIVP data, and input the data into the eCRF. The outcome assessor will obtain informed consent and collect preoperative as well as postoperative follow-up data. These data will be input into the eCRF by the data manager, who will log in using his or her own account information and who will be unable to access data input by the perfusionist. The statistician will see the two groups only as groups 1 and 2. The surgeon, perfusionist, and anesthesiologist will not be blinded to patient allocation because of the need to perform the corresponding procedures correctly. In cases when exclusion criteria are met after randomization (e.g., if total arch replacement is not performed), the patient will be withdrawn from the trial but will retain his or her identification code (randomization number).

Since the treatment allocation involves a surgical procedure, the surgeon, perfusionist, anesthesiologist, and other OR personnel will not be blinded to patient allocation. The perfusionist will record the intraoperative data. The assessor will be responsible for collecting preoperative data and obtaining informed consent, as well as for conducting postoperative visits. Physicians interacting with patients outside the OR will be blinded to treatment allocation. The details of the randomization will be kept confidential until completion of data analysis.

### Experimental intervention protocol

Patients will receive standard general anesthesia induction and maintenance with propofol, sevoflurane, sulfentanil, and muscle relaxants. All patients will be ventilated with a standard protocol (continuous mandatory ventilation, PCO_2_ 35–45 mmHg). Invasive blood pressures in the bilateral radial arteries, left dorsalis pedis artery, central venous pressure, and nasopharyngeal and rectal temperatures will be monitored. In addition, cerebral perfusion will be monitored using near-infrared spectroscopy (NIRS) throughout the surgery. Initial baseline cerebral tissue oxygen saturation will be obtained before anesthesia induction. A transesophageal echocardiographic probe will be routinely placed and used for confirmation of the diagnosis and assessment of cardiac function. A 1-mg/kg bolus of methylprednisolone will also be given.

The operations will be performed via a median sternotomy. After systemic heparinization (400 IU/kg), arterial cannulation (varied by surgeon preference using a central (distal ascending aorta, aortic arch, right axillary, or innominate arteries) or a peripheral (femoral artery) site for arterial cannulation under the guidance of ultrasound) and central cannulations of the superior and inferior venae cavae will be performed. The CPB circuit consists of a membrane oxygenator, a heat exchanger, and two rolling pumps, which can bifurcate the arterial line for both arterial perfusion and inferior vena caval perfusion as necessary [36]. The CPB will be primed with 1600–1800 mL solution (colloid and crystalloid in a 2:1 ratio with 5000 units of heparin). Systemic cooling will be initiated after CPB onset. CPB will be performed with a flow of 2.0–2.8 L/kg/min which will be adjusted to maintain a mixed venous oxygen saturation at around 85%. After the ascending aorta has been cross-clamped, cardiac arrest will be achieved with an infusion of cold-blood cardioplegia delivered in either antegrade or retrograde modes intermittently every 20 min. Acid-base balance will be maintained with the pH-stat method and a conventional modified ultrafiltration will be utilized during CPB.

During the cooling-down process, aortic root procedures, including repair of the aortic valve, or the Bentall procedure for severe aortic regurgitation and dilation of the aortic root, will be performed. Patients will undergo coronary artery bypass grafting if the coronary arteries are involved.

Patients will be systemically cooled to target nasopharyngeal temperature of 24–26 °C and target rectal temperature of 25–28 °C, then ACP or ACP + RIVP will be performed. For all patients, ACP will be established in a pressure-controlled manner (targeted pump pressure of 50–70 mmHg) using axillary cannulation, direct innominate artery cannulation, or left common carotid artery (flow rate at 5 to 10 mL/kg/min) to keep NIRS within 20% of baseline (over 50%). When the NIRS values decrease to < 50% or less than 80% of baseline, bilateral common carotid artery perfusion will be applied. At the initiation of ACP, the supra-aortic vessels will be gently clamped while the ascending aortic clamp and the aortic cannula are removed.

In control patients, only ACP will be performed and the lower body will not be perfused. In ACP + RIVP-group patients, RIVP will also be performed besides ACP. To achieve RIVP, the pump will be connected with the arterial line and inferior vena caval drainage to drive the blood from the arterial line to the inferior vena cava by cross-clamping of the drainage and placement of snares around the inferior vena caval cannula. RIVP will be performed at a flow rate of 5–12 mL/min/kg and perfusion pressure below 25 mmHg. Ascites can occur when capillary pressures are above this perfusion pressure cut-off [30]. During RIVP, an additional sucker will be then introduced into the proximal descending aorta to capture blood return during the reconstruction of the open distal anastomosis to maintain a bloodless field after the opening of the aortic arch.

During MHCA or RIVP, the arch will be resected distally to the orifice of the left subclavian artery with each head vessel prepared for individual anastomosis. A stent endograft (frozen elephant trunk) will be inserted into the true lumen of the descending aorta in an antegrade manner under direct vision. Then the aortic wall will be anastomosed to a four-branched graft end. After the distal anastomosis is completed and meticulous de-airing performed, RIVP will be stopped, and the systemic circulation through a side branch of the arch graft will be resumed to perfuse the lower body. Then the arch reconstruction will be carried out by anastomosis of the left common carotid artery, left subclavian artery and innominate artery with the three branches of the arch graft, respectively. ACP will stop, and rewarming will begin. After the anastomosis of the ascending aorta with the graft, the aortic clamp will be removed, and cardiac resuscitation will begin. Patients will be progressively weaned from CPB after the rectal temperature increases above 35.5 °C and the nasopharyngeal temperature reaches around 36.5 °C.

The remainder of the surgical procedure will be performed as standard, including hemostasis and closure of the incisions in layers. Blood components including packed red blood cells, fresh-frozen plasma, and platelets will be infused to maintain the post-CPB hematocrit and correct coagulopathy. Transfusion will be performed if hemoglobin concentration is < 7 g/dL during CPB, < 8 g/dL during surgery or < 9.5 g/dL in the ICU [37]. All patients will be managed in the ICU after the operation.

### Study outcomes

The primary outcome will be a composite of operative mortality and major complications, including paraplegia, postoperative renal failure, severe liver dysfunction, and gastrointestinal complications. Operative mortality is defined as any death that occurs in the same hospital in which the surgery was performed. Other complications will be defined according to the Society of Thoracic Surgery (https://www.sts.org/). The primary outcome will be measured throughout hospitalization (regardless of length of stay) and for up to 30 days after surgery if the patient is discharged. The decision to initiate renal replacement therapy (RRT) will be made by experienced staff in the cardiac ICU. Patients are given RRT if they are classified as grade F according to the RIFLE criteria: serum creatinine ≥ 4 mg/dL or an increase of at least threefold in baseline serum creatinine, or > 75% decline in the estimated glomerular filtration rate. Urine output < 0.3 mL/kg/h for 24 h or anuria for 12 h. We have defined RRT as intermittent hemodialysis or continuous venovenous hemofiltration [38].

Secondary outcomes will include the proportion of patients with a stroke/cerebrovascular incident, paraparesis, postoperative prolonged intubation (> 48 h), temporary neurological deficit, myocardial infarction (MI), acute kidney injury not requiring dialysis, surgical re-exploration for bleeding, and deep sternal wound infection. Patients who require assistance to stand or to walk will be defined as having paraparesis (Tarlov score 3–4) [39]. Temporary neurological deficit [40] will be defined as the occurrence of postoperative agitation, confusion, delirium, obtundation, or a transient focal neurological deficit (resolution within 72 h) without any evidence of new structural abnormality on computed tomography or magnetic resonance imaging. Myocardial infarction [41] will be defined as a rise and/or fall of cardiac troponin values at least one value above the 99th percentile upper reference limit and at least one of the following: symptoms of myocardial ischemia, new ischemic electrocardiogram (ECG) changes, development of pathological Q waves, imaging evidence of new loss of viable myocardium or new regional wall motion abnormality in a pattern consistent with an ischemic etiology, or the identification of a coronary thrombus by angiography or autopsy (but not for type 2 or 3 MI). Prolonged intubation will be defined as a requirement of intubation lasting more than 48 h.

Tertiary outcomes include the length of ICU stay, length of hospital stay, length of endotracheal intubation, volume of perioperative blood product transfusions, as well as total hospitalization cost. The proportion of patients who develop postoperative ascites will serve as a measure of safety in this trial.

### Monitoring of adverse and clinical events

Intraoperative data will include variables linked to the arterial cannulation site for cerebral perfusion (innominate artery, internal carotid artery, axillary artery, or subclavian artery), duration of HCA, CPB time, warming and cooling time, cross-clamp time, surgery time, temperature at the initiation of hypothermic circulatory arrest, concomitant procedures and the application of cross-clamp to the dissected aorta before initiation of hypothermic circulatory arrest, number of units of packed red blood cells, fresh-frozen plasma, pooled platelets and cryoprecipitate administered perioperatively, the highest lactate value during CPB, and the highest flow and pressure of ACP and RIVP.

Patients will be monitored on a daily basis for 7 days after surgery to collect data on temperature, partial pressure of oxygen (PaO_2_) and carbon dioxide (PaCO_2_), inspired oxygen (FiO_2_), ventilation mode, hemoglobin and leukocyte count, and volume of chest drainage. Symptomatic cardiorespiratory complications and other secondary outcome measures (see above) will also be recorded during routine diagnostic tests. These parameters and the timing of notable events will be tracked until hospital discharge.

Patients will be instructed to visit during the hospitalization or following discharge for postoperative data collection. If a face-to-face appointment is not possible, follow-up will be completed over the phone. During each visit, patient characteristics, including mortality, cardiovascular and cerebrovascular events, postoperative renal and liver function, and gastrointestinal complications, will be recorded, Abdominal ultrasound will also be performed at 7 days postoperatively. In addition, radiology and electrocardiography will be performed at each visit after discharge, uploaded to the database and evaluated by outcome assessors blinded to patient allocation.

Preoperative and postoperative data will be recorded by a member of the research team at each participating center who will be blinded to randomization status and who will not be part of the surgical team that performs the interventions. Intraoperative data will be collected by the study perfusionists and anesthesiologists.

### Safety and monitoring

An independent data and safety monitoring board comprising cardiovascular surgeons, anesthesiologists and statisticians will oversee the progress and safety of the study, including adverse events and morbidity. All adverse events will be evaluated for severity. Any severe adverse events will be recorded on the CRF and reported within 24 h to the Board and the Biological and Medical Ethics Committee of West China Hospital.

All unexpected major cardiovascular, cerebrovascular, and other serious adverse events not listed in the protocol will be reported to the coordinating center within 24 h. The chief principal investigator will be responsible for all adverse event reporting. All adverse events will be closely followed until resolution or stabilization. A local investigator will review all reports of adverse events.

### Data management and quality control

All CRFs will be immediately entered into a secure, web-based system hosted by the Data Coordinating Center as soon as they are received. Designated research team members will be authorized to access the allocation system and electronic CRFs by entering the patient’s unique participant identification number, initials, and date of birth in an online form. If data are entered incompletely or incorrectly, the principal investigator will contact the participating centers for clarification.

To control the quality of this study, all perfusionists will receive centralized training before the trial begins. All stored records will be kept secure and confidential according to standard guidelines. A reason must be indicated whenever data are altered, and all alterations will be saved.

### Sample size calculation

The sample size was calculated based on the primary outcome of our unpublished pilot study (manuscript in preparation), carried out at West China Hospital (Biomedical Ethics Committee approval no. 201824). In this study, we included 76 patients (*n* = 38 in each group), and defined the primary outcome as the abovementioned 30-day composite of operative mortality and major complications. In this pilot study, 13 of 38 control patients (34.2%) suffered the following outcomes: death, 4; paraplegia, 1; postoperative renal failure, 7; severe liver dysfunction, 4; or gastrointestinal complications, 1. A total of 9 of 38 patients in the RIVP group (23.7%) experienced the following outcomes: death, 3; paraplegia, 0; postoperative renal failure, 6; severe liver dysfunction, 1; or gastrointestinal complications, 1. The trial is planned to have 80% test power with a two-sided type I error rate of 5%, assuming a dropout rate of 10% over the course of the entire study. For statistical significance, 636 patients (318 participants in each arm) will be required for this trial according to the following statistical formula not including the 76 patients in the pilot study:

$$ \mathrm{n}=\frac{{\left({Z}_{1-\alpha /2}+{Z}_{1-\beta}\right)}^2\left[{p}_1\left(1-{p}_1\right)+{p}_2\left(1-{p}_2\right)\right]}{\delta^2} $$

### Data analysis

All patients will be analyzed according to the intention-to-treat protocol. Data analysis will be performed by a statistician using SPSS 20.0 (IBM, Chicago, IL, USA). Differences associated with *p* < 0.05 will be considered statistically significant. Continuous variables will be expressed as mean ± standard deviation or median (interquartile range), and differences in such variables will be analyzed using an independent *t* test or the Wilcoxon signed-rank test, depending on whether data are normally distributed. Categorical variables will be described as numbers (percentages), and differences in such variables will be analyzed using a *chi-square test* and *Fisher’s exact test*. *Kaplan-Meier* curves and *log-rank* analysis will be used to compare inter-group differences in primary and secondary outcomes. Univariate and multivariate logistic regression will be performed to determine relative risk of primary and secondary outcomes in the RIVP group compared with the control group.

### Timeline

- 2018–2019: Development of research strategy and study protocol.
- 2019–2021: Recruitment and treatment of patients in RIVP-TARS trial.
- 2021–2022: Completion of follow-up and data analysis.

## Discussion

### Trial rationale

Improvements in CPB strategies, including selective cerebral perfusion and temperature management, have led to lower incidence of neurological dysfunction and other complications during the period of circulatory arrest than with DHCA. However, few studies have examined CPB improvement strategies aimed at visceral and lower-body perfusion. We and others propose that maintaining the continuity of lower-body blood flow may be important for avoiding visceral organ dysfunction and postoperative mortality [42, 43].

Distal perfusion through the descending aorta or femoral artery has previously been used to minimize damage to organs [44, 45]. However, this technique is associated with risk of poor occlusion, which seriously interferes with the operating field and leads to false lumen perfusion during femoral artery cannulation. Increasing perfusion through the inferior vena cava may help preserve visceral function and improve TARS outcomes, especially since the viscera lack venous valves.

Our trial will combine ACP and RIVP during TARS using independently controlled upper- and lower-body perfusion circuits. This trial is expected to provide up-to-date data on the safety and efficacy of RIVP in patients undergoing TARS and has the potential to reduce the incidence of circulatory-arrest-associated complications and perioperative blood product transfusion in patients with AAAD, ultimately improving long- and short-term prognosis.

### Trial status

We have completed the eCRF system. The study opened to patient recruitment in January 2019. Completion of this trial is expected on 31 December 2021.

## Additional file

Additional file 1: SPIRIT 2013 Checklist: Recommended items to address in a clinical trial protocol and related documents. (DOC 126 kb)

## Abbreviations

AAAD: Acute type A aortic dissection
ACP: Selective antegrade cerebral perfusion
CONSORT: Consolidated Standards of Reporting Trials
CPB: Cardiopulmonary bypass
CRF: Case report form
DHCA: Deep hypothermic circulatory arrest
eCRF: Electronic case report form
EuroSCORE II: European System for Cardiac Operative Risk Evaluation II
FiO_2_: Fraction of inspired oxygen
HCA: Hypothermic circulatory arrest
ICU: Intensive care unit
MHCA: Moderate hypothermic circulatory arrest
MI: Myocardial infarction
PaCO_2_: Partial pressure of carbon dioxide
PaO_2_: Partial pressure of oxygen
RCP: Retrograde cerebral perfusion
RIVP: Retrograde inferior vena caval perfusion
RRT: Renal replacement therapy
SPIRIT: Standard Protocol Items: Recommendations for Interventional Trials
TARS: Total aortic arch replacement surgery
